# One-year mortality and morbidities of severe fever with thrombocytopenia syndrome compared with other diseases: A nationwide cohort study in South Korea

**DOI:** 10.1371/journal.pntd.0012253

**Published:** 2024-06-14

**Authors:** Namwoo Heo, Seok-Jae Heo, Yoon Soo Park, Seonju Yi, Hyunju Lee, Hyo-jung Lee, Yong Chan Kim

**Affiliations:** 1 Division of Infectious Disease, Department of Internal Medicine, Yongin Severance Hospital, Yonsei University College of Medicine, Yongin, Republic of Korea; 2 Division of Biostatistics, Department of Biomedical Systems Informatics, Yonsei University College of Medicine, Seoul, Republic of Korea; 3 Korea Disease Control and Prevention Agency, Cheongju, Republic of Korea; 4 Department of Prevention Medicine, Seoul National University College of Medicine, Seoul, Republic of Korea; Instituto de Medicina Tropical - USP, BRAZIL

## Abstract

**Background:**

The long-term mortality and morbidity of patients with severe fever with thrombocytopenia syndrome (SFTS) remain unclear.

**Methods:**

This retrospective cohort study was conducted using the National Health Insurance Service dataset on hospitalized patients with SFTS aged ≥20 years between 2016 and 2021 (n = 1,217). Each SFTS case was matched with three controls hospitalized for non-SFTS-related diseases using propensity score matching. The all-cause mortality of patients with SFTS was evaluated during the one-year follow-up and compared with that of controls. Post-discharge events were investigated to determine the effects of SFTS on post-acute sequelae.

**Results:**

Finally, 1,105 patients with SFTS and 3,315 controls were included. Patients with SFTS had a higher risk of death during the one-year follow-up than that of controls (hazard ratio [HR], 2·26; 95% confidence interval [CI], 1·82–2·81). Thirty-day mortality was significantly higher in the SFTS group (HR, 3·99; 95% CI, 3·07–5·19) than in the control group. An increased risk of death after 31–365 days was observed among controls, though this difference was significant only among patients in their 80s (HR, 0·18; 95% CI, 0·06–0·57). For post-discharge events, patients in the SFTS group exhibited a higher risk of readmission (HR, 1·17; 95% CI, 1·04–1·32) and emergency room visit (HR, 2·32; 95% CI, 1·96–2·76) than those in the control group.

**Conclusion:**

SFTS induces a higher risk of short-term mortality and post-acute sequelae in hospitalized patients during a one-year follow-up than non-SFTS-related diseases. Our results provide guidance for the management of SFTS.

## Author summary

Severe fever with thrombocytopenia syndrome (SFTS) is a life-threatening viral disease, which has expanded beyond Asian countries. However, most studies have focused on the short-term mortality of patients with SFTS, and knowledge of their long-term outcomes is limited. This nationwide cohort study evaluated long-term outcomes of hospitalized patients with SFTS between 2016 and 2021 using the national dataset. We found that hospitalized patients with SFTS had higher one-year mortality (HR, 2.26) than those without SFTS. An increased mortality risk was attributed to death in the first 30 days of hospitalisation (HR, 3.99). Although the long-term mortality (31–365 days) did not differ between patients with and without SFTS, except for those in their 80s, patients with SFTS suffered from post-acute events, including readmission (HR, 1.17) or emergency room visit (HR, 2.32) during the one-year follow-up. SFTS is an emerging infectious disease that demands attention. The disease burden extends beyond the acute phase, with patients often experiencing ongoing health problems even after hospital discharge. Our findings underscore the post-acute burden of SFTS and offer valuable insights for the development of effective management plans for acute and post-acute illnesses in patients with SFTS.

## Introduction

Severe fever with thrombocytopenia syndrome (SFTS) is an infectious disease caused by *Dabie bandavirus*, which belongs to the genus *Bandavirus* in the family *Phenuiviridae* and order *Bunyavirales* [1]. Ticks primarily transmit bandavirus; however, some studies have reported bodily fluids as the source of infection [2,3]. The main symptoms of SFTS include fever, thrombocytopenia, and leukopenia [4,5]. Some patients progress to severe illnesses, including neurological symptoms and multiple organ failure [1,5]. However, we currently lack specific therapy for SFTS [4].

SFTS was first reported in rural areas of Hubei and Henan provinces in China in 2009 [1]; it has since been continuously reported in various Asian countries, including Korea, Japan and Vietnam [6–9]. Currently, SFTS is considered an emerging infectious disease that requires the attention of residents and travelers in the aforementioned regions. In South Korea, it was designated as a mandatory notifiable infectious disease in 2013 [10]. The number of reported cases demonstrates an increasing trend every year, exceeding 100 for the first time in 2016 and reaching 272, the highest number of cases thus far in 2017 [10]. Although most cases occur sporadically in the community, some cases of nosocomial transmission caused by contact with acutely ill patients with SFTS have been reported [3,11].

The mortality rate of SFTS is approximately 6–32%, with some differences occurring between studies [12–15]. However, to date, most studies have focused on the short-term outcomes of patients with SFTS during hospitalization [14–16], and we lack studies on long-term outcomes. Understanding the long-term outcomes is essential to gauge the prognosis of SFTS, predict disease burden, and minimize the sequelae of this infection. Therefore, we analyzed the long-term outcomes of SFTS during a one-year follow-up period among hospitalized patients with SFTS by comparing them with those hospitalized for other diseases using nationwide big data.

## Methods

### Ethics statement

This study was approved by the Institutional Review Board of the Yonsei University College of Medicine, Yongin Severance Hospital (approval number: 9-2022-0084), which waived the requirement for informed consent owing to the retrospective and deidentified nature of the study.

### Study design and data source

We conducted a retrospective cohort study to evaluate the effect of SFTS on one-year mortality and morbidity of patients using the National Health Insurance System (NHIS) dataset in South Korea. The NHIS dataset, which covers approximately 98% of the Korean population, is ideal for national-level big data research because it is a universal medical security system that covers various types of data ranging from disease diagnoses to personal information and can be combined with various sources of national data [17].

### Study population

The study population comprised hospitalized patients aged ≥20 years who were diagnosed with SFTS and non-SFTS-related diseases at the time of hospitalization between 1 January 2016 and 31 December 2021. Since SFTS was first coded by the Korean Standard Classification of Diseases in January 2016, we reviewed all cases of SFTS from that point onward. For patients with multiple hospitalizations, only data from the first hospitalization was included to avoid the duplicate assessment of the same patient.

Patients with SFTS were defined as those with a diagnostic code (Korean Standard Classification of Diseases and Causes of Death, 7–8^th^ edition [KCD-7,8 code]) A93·80 for the first time. During the study period, SFTS was diagnosed according to guidelines provided by the Korea Disease Control and Prevention Agency (KDCA). SFTS diagnosis was confirmed according to the following criteria: patients with (1) clinical manifestations of thrombocytopenia with leukopenia, gastrointestinal symptoms, such as nausea and vomiting, lymphadenopathy, neurologic symptoms, organ dysfunction, and high fever (38–40°C) for 3–10 days without other causes and (2) isolation of SFTS virus from the blood sample, an antibody level with ≥four-fold increase between paired serum samples, or a positive polymerase chain reaction result for SFTS virus. In South Korea, once a diagnosis of SFTS is confirmed, the corresponding case is also investigated by the KDCA and local public health centers. Therefore, the chances of misdiagnosis or overestimation are low.

For each case of SFTS, we recruited control patients from the NHIS dataset who were admitted to hospitals owing to non-SFTS-related diseases during the same month of the same year. The control patients were alive at the time of admission and had not been diagnosed with SFTS before they were matched.

### Covariates

We collected information on sex, age group (10-year age bands), the month of hospitalization, insurance type, household income level, intensive care unit (ICU) admission during the first seven days of hospitalization, previous hospitalization history within one year before the event that led to admission, and pre-index comorbidities as covariates. Household income was classified into low (T1), middle (T2), and high (T3) levels. Disease severity at the time of hospitalization was determined based on the history of ICU admission within seven days of hospitalization. Underlying comorbidities were analyzed based on data obtained within three years before admission. The following comorbidities were selected: hypertension, diabetes mellitus, stroke, heart failure, atrial fibrillation, coronary artery occlusive disease, asthma, chronic kidney disease, and malignancy [18]. We used the KCD-7,8 codes, a modified version of the International Classification of Diseases, 10^th^ revision, to define these diseases (**S1 Table**).

### Outcomes

The main outcome of this study was all-cause mortality in patients with SFTS after hospitalization during the one-year follow-up compared with that in patients without SFTS (controls). For all participants in this study, the follow-up period was from the time of hospitalization to the end of the one-year observation period or day of death. We also investigated post-discharge events, including readmission, emergency room (ER) visits, cardiovascular diseases, cerebrovascular events, and ICU admission after discharge as secondary endpoints.

### Propensity score matching (PSM)

PSM was performed to balance the baseline characteristics using the greedy nearest-neighbor algorithm at a ratio of 1:3. Propensity scores were calculated using logistic regression with all available covariates, and the months of hospitalization were exactly matched. Standardized mean differences (SMDs) were used to determine the balance between groups before and after matching. When the SMD value was <0·1, distribution was considered to have a good balance between the SFTS and control groups.

### Statistical analysis

All covariates are presented as mean±standard deviation for continuous variables or frequency (percentage) for categorical variables. The Kaplan–Meier curve was used to calculate the survival probabilities and cumulative incidence. The Cox proportional hazard model was used to estimate hazard ratios (HRs) and 95% confidence intervals (CIs) for the one-year mortality and morbidity rates. For the primary outcome, subgroup analyses were conducted for covariates, including sex, household income level, comorbidities, previous hospitalization, and severity at admission (ICU admission). All statistical analyses were conducted using the R software (version 4·2·0; The R Foundation for Statistical Computing, Vienna, Austria). A p-value <0·05 was considered statistically significant.

## Results

### Baseline characteristics of patients

From 1 January 2016 to 31 December 2021, 1,217 patients were hospitalized with SFTS **(Table 1)**. Moreover, 1,136,306 patients hospitalized for non-SFTS-related diseases during the same period were randomly selected from the NHIS dataset.

**Table 1:** Baseline characteristics of patients hospitalized with SFTS or non-SFTS-related diseases between 2016 and 2021.

Variable	Before PSM			After PSM		
	SFTS	Control	SMD	SFTS	Control	SMD
Total	1,217	1,136,306		1,105	3,315	
Sex, n (%)			0.012			0.038
Male	582 (47.8)	536,568 (47.2)		521 (47.2)	1,626 (49.1)	
Female	635 (52.2)	599,738 (52.8)		584 (52.9)	1,689 (51.0)	
Age group, n (%)			1.018			< .001
20~49	103 (8.5)	315,954 (27.8)		100 (9.1)	300 (9.1)	
50~59	229 (18.8)	229,116 (20.2)		216 (19.6)	648 (19.6)	
60~69	333 (27.4)	266,335 (23.4)		304 (27.5)	912 (27.5)	
70~79	347 (28.5)	199,275 (17.5)		309 (28.0)	927 (28.0)	
80~	205 (16.8)	125,626 (11.1)		176 (15.9)	528 (15.9)	
Insurance type, n (%)			0.176			0.044
National health insurance	1177 (96.7)	1,054,709 (92.8)		1,067 (96.6)	3,173 (95.7)	
Medical aid	40 (3.3)	81,597 (7.2)		38 (3.4)	142 (4.3)	
Household income, n (%)			0.175			0.056
T1, lowest	284 (23.3)	329,162 (29.0)		266 (24.1)	726 (21.9)	
T2	364 (29.9)	369,664 (32.5)		324 (29.3)	1,030 (31.1)	
T3, highest	569 (46.8)	437,480 (38.5)		515 (46.6)	1,559 (47)	
Comorbidities, n (%)						
Hypertension	612 (50.3)	615,272 (54.2)	0.077	554 (50.1)	1,662 (50.1)	< .001
Diabetes mellitus	477 (39.2)	611,897 (53.9)	0.297	428 (38.7)	1,313 (39.6)	0.018
Stroke	117 (9.6)	175,345 (15.4)	0.176	87 (7.9)	261 (7.9)	< .001
Heart failure	151 (12.4)	266,997 (23.5)	0.292	106 (9.6)	318 (9.6)	< .001
Atrial fibrillation	37 (3.0)	70,612 (6.2)	0.152	32 (2.9)	92 (2.8)	0.007
Coronary artery occlusive disease	195 (16.0)	326,157 (28.7)	0.308	151 (13.7)	453 (13.7)	< .001
Asthma	228 (18.7)	378,753 (33.3)	0.337	200 (18.1)	594 (17.9)	0.005
Chronic kidney disease	70 (5.8)	149,392 (13.2)	0.255	60 (5.4)	185 (5.6)	0.007
Malignancy	134 (11.0)	402,638 (35.4)	0.604	112 (10.1)	311 (9.4)	0.025
ICU admission within 7days after hospitalization, n (%)	425 (34.9)	99,052 (8.7)	0.669	323 (29.2)	969 (29.2)	< .001
Previous hospitalization days within 1 year, mean (±SD)	14.5 ± 15.5	43.2 ± 70.5	0.408	13.6 ± 12.1	14.4 ± 14.1	0.057

Abbreviations: SFTS, severe fever with thrombocytopenia syndrome; PSM, propensity score matching; SMD, standardized mean difference; T, tertile; ICU, intensive care unit; SD, standard deviation.

Before PSM, among those who were hospitalized for SFTS, 47·8% (n = 582) were men; patients in their 70s and 60s comprised 28·5% (n = 347) and 27·4% (n = 333) of the entire population, respectively, indicating a high representation of older individuals. In the control group, 47·2% (n = 536,568) of patients were men, and most were aged 20–49 years (27·8%, n = 315,954). The percentage of patients with national health insurance in the SFTS and control groups was 96·7% (n = 1,177) and 92·8% (n = 1,054,709), respectively. When examining household income levels, the T3 group, the highest-income group, occupied the largest percentage in both groups. The most common comorbidity in both groups was hypertension, followed by diabetes mellitus. The ICU admission rate during the first seven days after hospitalization, which represents disease severity, was 34·9% in the SFTS group, which was considerably higher than that in the control group (8·7%). In contrast, the number of days hospitalized within one year before the event that led to admission was higher in the control group (43·2±70·5 days) than in the SFTS group (14·5±15·5 days).

After PSM, we identified 1,105 patients with SFTS and 3,315 patients with other diseases as well-matched pairs. Overall, there were no significant differences in baseline characteristics between the SFTS and control groups after PSM, with an SMD of <0·1 for all covariates.

### Mortality

One-year mortality rates of patients with SFTS or non-SFTS-related diseases are presented in **Table 2**. Among patients hospitalized for SFTS, 152 died during the first year after hospitalization (158·9 per 1,000 patient-years), whereas 238 patients died during the same period in the control group (76·5 per 1,000 patient-years), indicating that the SFTS group had a higher risk of death than had the control group (HR, 2·261; 95% CI, 1·823–2·806; p<0·001). During this period, the average time from the first day of hospitalization to death in the SFTS group was 20·8±52·4 days, much shorter than that of the control group (76·2±93·3 days) (**S2 Table**).

**Table 2:** One-year mortality and morbidities in patients hospitalized with SFTS or non-SFTS-related diseases.

Outcome	Event		Event per 1,000 patient-years		HR (95% CI)	p-value
	SFTS	Control	SFTS	Control		
Death						
0–365 days mortality	152	238	158.9	76.5	2.261 (1.823–2.806)	<0.001
0–30 days mortality	134	118	1626.4	444.0	3.988 (3.066–5.186)	< .001
31–90 days mortality	10	46	42.0	58.8	0.780 (0.383–1.590)	0.495
91–180 days mortality	1	32	2.1	20.7	0.108 (0.015–0.801)	0.030
181–365 days mortality	6	39	6.3	12.6	0.445 (0.183–1.080)	0.074
Events after discharge						
Hospital admission	424	1256	659.6	561.1	1.170 (1.037–1.320)	0.011
ER visit	251	422	327.8	149.4	2.324 (1.960–2.755)	< .001
Cardiovascular disease	163	496	195.8	183.0	1.120 (0.924–1.358)	0.249
Cerebrovascular event	142	619	168.1	236.4	0.733 (0.602–0.893)	0.002
ICU admission	11	35	11.6	11.3	0.929 (0.461–1.872)	0.837

Abbreviations: SFTS, severe fever with thrombocytopenia syndrome; HR, hazard ratio; CI, confidential interval; ER, emergency room; ICU, intensive care unit.

Mortality occurred at higher concentrations within the first 30 days in both groups. During this period, the mortality rate was significantly higher in the SFTS group (1626·4 per 1,000 patient-years) than in the control group (444·0 per 1,000 patient-years) (HR, 3·988; 95% CI, 3·066–5·186; p<0·001). The time from the first day of hospitalization to death in this period (0–30 days) was also shorter in the SFTS group than in the control group (7·0±5·4 vs 9·2±7·9 days). The mortality between 31–365 days was observed at 128·6 ± 108·5 days in SFTS patients and 144·9 ± 91·3 days in the control group (**S2 Table**).

In contrast, when analyzing deaths from 31 to 365 days, the mortality rate was higher in the control group than in the SFTS group (HR, 0·478; 95% CI, 0·283–0·807; p = 0·006). Even when the period of death after 31 days was further subdivided (31–90, 91–180, and 181–365 days), mortality tended to be lower in the SFTS group than in the control group. A significant difference was only observed at 91–180 days (HR, 0·108; 95% CI, 0·015–0·801; p = 0·030), though statistical significance was observed in the 31–90-day (HR, 0·780; 95% CI, 0·383–1·590; p = 0·495) or 181–365-day (HR, 0·445; 95% CI, 0·183–1·080; p = 0·074) periods. As for the time to death at 31–365 days, the patients in the SFTS group had a shorter survival time (128·6±108·5 days) than those in the control group (144·9±91·3 days).

**Fig 1:**
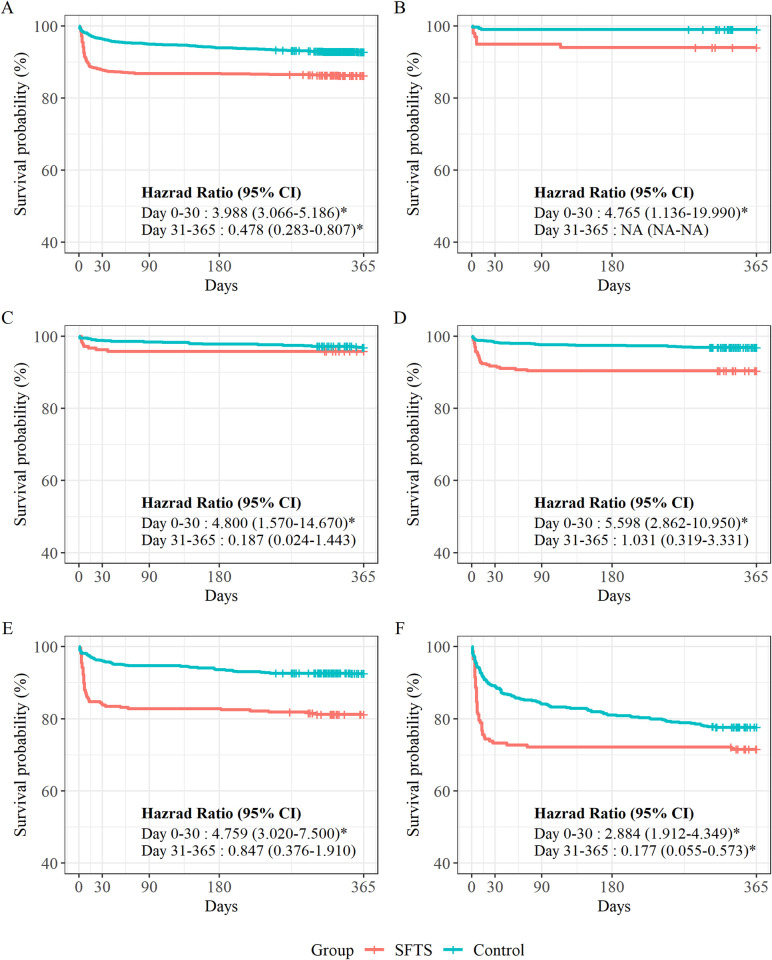
Age-specific Kaplan–Meier curves and hazard ratios for patients with SFTS or non-SFTS-related diseases. (A) All age groups; (B) patients aged 20–49 years; (C) patients aged 50–59 years; (D) patients aged 60–69 years; (E) patients aged 70–79 years; and (F) patients aged 80–89 years. The disaggregated results for 0–30 and 31–365 days are reported in **S1 and S2 Figs**.

In the Kaplan-Meier survival analysis (**Fig 1**), the hazard ratio for the first 30 days in the SFTS group was higher in all age groups, with the highest risk among patients in their 60s and the lowest risk among patients in their 80s. Among patients in their 80s, mortality at 31–365 days was significantly lower in the SFTS group than in the non-SFTS group (HR, 0·177; 95% CI, 0·055–0·573), and there was no statistically significant difference in other age groups.

### Post-discharge events other than deaths

**Fig 2:**
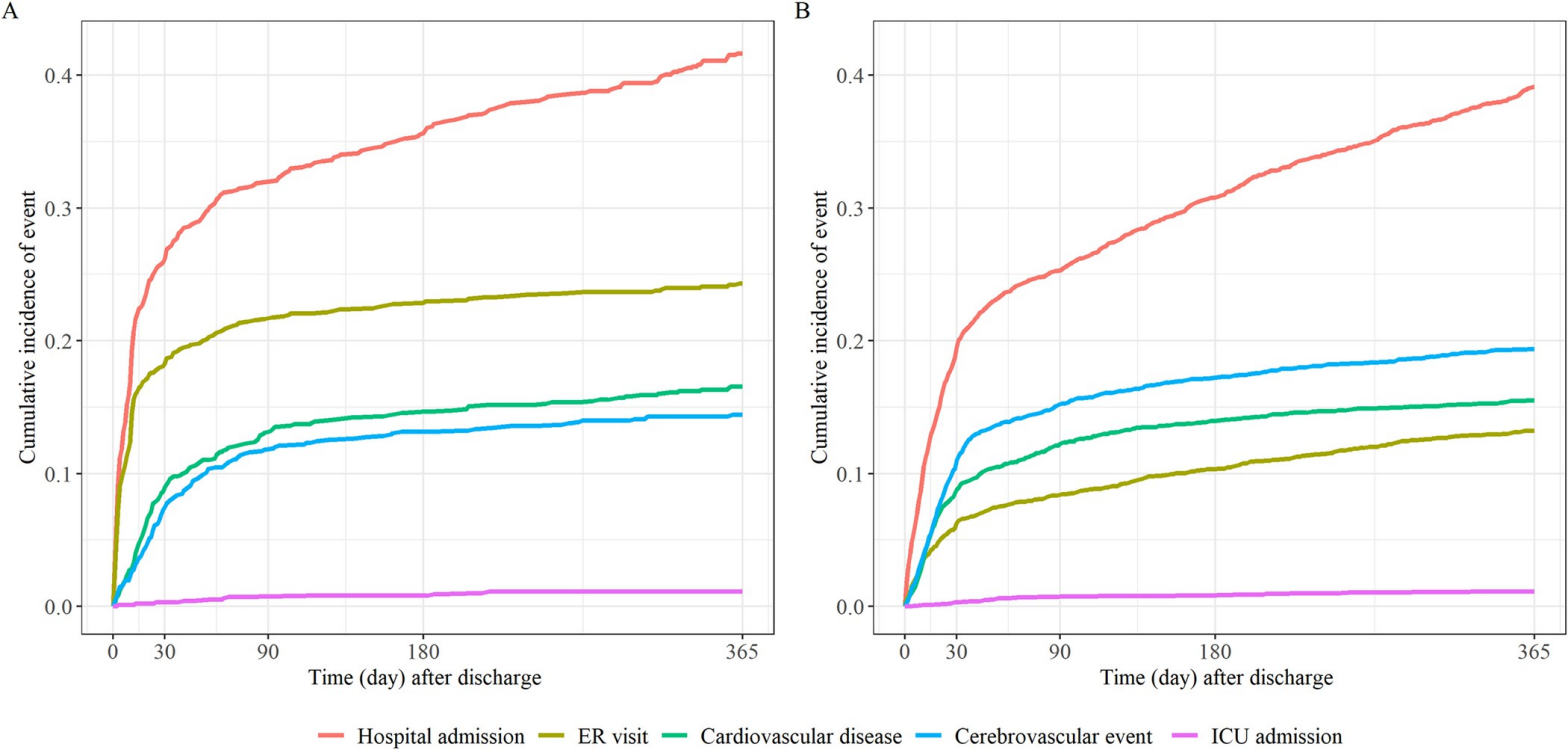
**Cumulative incidence of post-discharge events during the one-year follow-up** (A) SFTS and (B) non-SFTS-related disease groups. The disaggregated results for 0–30 and 31–365 days are displayed in **S3 Fig**, results by age group are displayed in **S4 Fig**.

The risk of readmission after discharge (HR, 1·170; 95% CI, 1·037–1·320; p = 0·011) and ER visits after discharge (HR, 2·324; 95% CI, 1·960–2·755; p<0·001) was higher in the SFTS group than in the control group (**Table 2**). The time from discharge to readmission was shorter in the SFTS group (60·2±93·7 days) than in the non-SFTS group (87·6±104·3 days) (**Fig 2 and S3 Table**). The time taken from discharge to visiting the ER was also shorter in the SFTS group (33·9±67·5 days) than in the non-SFTS group (88·5±100·8 days). Conversely, there was no statistically significant difference between both groups in terms of admission to the ICU after discharge: The time taken from discharge to ICU admission was 90·7±80·0 days and 96·5±92·5 days in the SFTS and control groups, respectively.

There was no significant difference in the risk of cardiovascular disease after discharge between both groups; however, the risk of cerebrovascular events was lower in the SFTS group than in the control group (HR, 0·733; 95% CI, 0·602–0·893; p = 0·002).

### Subgroup analysis

**Fig 3:**
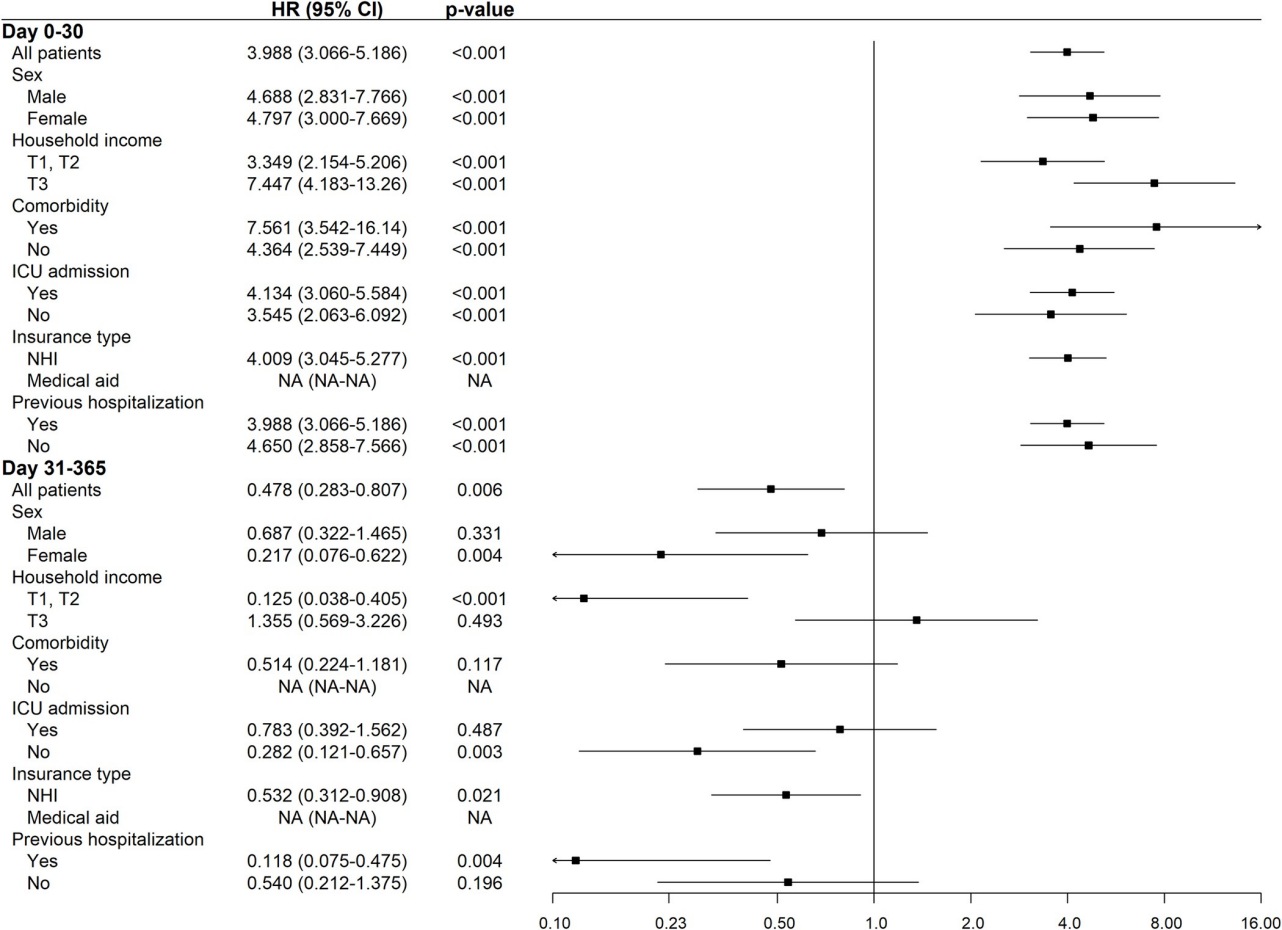
Hazard ratios in various subgroups. For early (day 0–30) and late (day 31–365) mortality in patients with SFTS or non-SFTS-related diseases.

When the primary outcome was analyzed in several subgroups (**Fig 3**), the risk of death within 30 days of hospitalization was higher in the SFTS group, regardless of sex, household income level, comorbidity, insurance type, previous hospitalization, and severity at admission (ICU admission). Women (HR, 0·217; 95% CI, 0·076–0·622; p = 0·004) and patients with low household income (T1, T2) (HR, 0·125; 95% CI, 0·038–0·405; p<0·001), no ICU admission (HR, 0·282; 95% CI, 0·121–0·657; p = 0·003), national health insurance (HR, 0·532; 95% CI, 0·312–0·908; p = 0·021), and previous hospitalization history (HR, 0·118; 95% CI, 0·075–0·475; p = 0·004) had a significantly higher risk of mortality at 31–365 days than did the remaining participants.

## Discussion

To our knowledge, this is the first study to investigate the long-term impact of SFTS compared with non-SFTS-related diseases at a one-year follow-up. Healthcare providers usually face difficulties in clinical decision-making regarding which patients are more likely to have a severe course and require immediate hospitalization and how long they should be followed up with care after discharge. Our results provide evidence for the management of patients with SFTS.

Although SFTS cases have been reported across South Korea, most occur in grassy, brushy, or wooded areas suitable for ticks, including Gyeonggi, Gangwon, and Gyeongbuk [19]. Notably, agricultural farmers, who have occupational risk factors associated with SFTS [12,20,21], are most likely to be exposed to ticks infected with *Dabie bandavirus*. Considering that older people account for a large proportion of agricultural farmers in South Korea, the age distribution of patients with SFTS in this study was concentrated in the older age group. The predominance of older adults is also evident in epidemiological studies in Japan and China [13–15].

Concordant with the results in several previous studies [12,15,16], we also demonstrated a high risk of death within 30 days, which was four times higher in the SFTS group than in the control group; this risk of short-term mortality was observed in all age groups. Such high short-term mortality in SFTS may be associated with a cytokine storm, a life-threatening systemic inflammatory syndrome [22]. *Dabie bandavirus* infection triggers an immune response shortly thereafter. However, this response to the pathogen sometimes results in immune-cell hyperactivation and highly elevated cytokine levels, which can cause multiple organ damage. Our hypothesis regarding the exact pathogenesis of SFTS should be validated in future studies.

In contrast to short-term mortality, an increased risk of death after 30 days was observed among patients in the control group, although this was only statistically significant in patients in their 80s. Due to the fatal outcomes within one month of SFTS, it can be hypothesized that only those patients who are less fragile survived following the infection, compared to those in the control group. However, patients in the SFTS group had a higher chance of readmission or ER visits after discharge than those in the control group, indicating that patients with SFTS may experience post-acute sequelae other than death. Prospective observational cohort studies should be conducted to identify in more detail the complications experienced by patients with SFTS who have passed the acute phase of illness.

Encephalitis or encephalopathy was diagnosed in 19% of patients hospitalized for SFTS [23], and central nervous system (CNS) involvement in SFTS resulted in a high mortality rate. According to one study, 44% of patients diagnosed with encephalitis died [23]. CNS manifestations in SFTS are associated with the involvement of *Dabie bandavirus*, which can cause cerebrovasculopathy [24]. Cerebrovascular inflammation caused by viral infections can result in cerebrovascular events. Varicella zoster virus infection is known to increase the risk of stroke in patients through CNS infections and subsequent cerebrovasculopathy [25]. However, as presented in our study, the risk of post-discharge cerebrovascular events was lower in patients with SFTS than in controls. Since patients with SFTS and CNS manifestations have a high risk of death [5], many die during hospitalization, which may reduce the number of observable cases of cerebrovascular events after discharge. To accurately predict cerebral vascular remodeling triggered by the SFTS virus and its effects, further studies conducted among patients with SFTS and CNS symptoms are required.

Currently, our knowledge of the convalescent symptoms in patients with SFTS remains limited. Hematospermia, fatigue, myalgia, alopecia, insomnia, and depression were reported during the recovery period of patients with SFTS in a case study in Japan [26]. Whether these symptoms are 1) a long-term complication occurring after critical care; 2) viral hemorrhagic fever-specific post-acute sequelae, as seen with post-Ebola syndrome; or 3) a post-infection condition that can also be observed in other viral infections, such as severe acute respiratory syndrome coronavirus 2 infection, remains unclear [27–29].

This study had some limitations. First, owing to the retrospective nature of the study, we could not control for all confounding factors that could have affected the outcome. Particularly, while we attempted to select the control from the same hospitals as the group of patients with SFTS to reduce the impact of regional differences in medical standards, we were unable to obtain hospitals information from the NHIS due to the possibility of patient identification, preventing us from matching the patient groups in the same hospital. However, the most critical variables that can affect outcomes were well-controlled, thereby facilitating a proper comparison between SFTS and non-SFTS-related diseases. Second, although our study included almost all Korean residents, some patients with SFTS were likely not included; thus, there may have been a selection bias. For example, patients excluded from the study may have included individuals who died before diagnosis or were not tested by the medical staff because of vague symptoms and signs. Third, while we presumed that almost all deaths of patients with SFTS would have been attributed to SFTS itself, we were unable to determine the exact cause of death because we could not secure the cause of death data for individual cases. Fourth, despite examining the post-acute burden of SFTS by comparing ICU admissions within 7 days after hospitalization to the control group, the possibility of inability to verify the specific severity of the diseases remains. Finally, because this study included only cases that occurred in South Korea, the generalizability of the results of this study in other countries with different medical environments may be limited.

In conclusion, an increased risk of death was observed in the short term, but not in the long term, in patients with SFTS compared with those with non-SFTS-related disease, regardless of their age. However, SFTS continues to affect the daily lives of patients after discharge from the hospital, highlighting the post-acute burden of the disease. Our findings regarding the disease course of SFTS over time may help design management plans for acute and post-acute illnesses in patients with SFTS.

## Supporting information

S1 Table KCD-7, 8 codes for comorbidities. (DOCX)

S2 Table Time from hospitalization to death in patients with SFTS or non-SFTS-related diseases. (DOCX)

S3 Table Time from discharge to events in patients with SFTS or non-SFTS-related diseases. (DOCX)

S1 Fig **Age-specific Kaplan–Meier curves and hazard ratios for patients with SFTS or non-SFTS-related diseases for 0–30 days after hospitalization** (A) All age groups; (B) patients aged 20–49 years; (C) patients aged 50–59 years; (D) patients aged 60–69 years; (E) patients aged 70–79 years; and (F) patients aged 80–89 years.
(DOCX)

S2 Fig **Age-specific Kaplan–Meier curves and hazard ratios for patients with SFTS or non-SFTS-related diseases for 31–365 days after hospitalization** (A) All age groups; (B) patients aged 20–49 years; (C) patients aged 50–59 years; (D) patients aged 60–69 years; (E) patients aged 70–79 years; and (F) patients aged 80–89 years.
(DOCX)

S3 Fig **Cumulative incidence of post-discharge event during the one-year follow-up** in the (A) SFTS group at 0–30 days, (B) SFTS group at 31–365 days, (C) non-SFTS-related disease group at 0–30 days, and (D) non-SFTS-related disease group at days 31–365.
(DOCX)

S4 Fig **Cumulative incidence of post-discharge event during the one-year follow-up** in patients with SFTS (A, C, E, G, and I) and non-SFTS patients (B, D, F, H, and J). (A, B) aged 20–49 years, (C, D) aged 50–59 years, (E, F) aged 60–69 years, (G, H) aged 70–79 years, (I, J) aged 80–89 years.
(DOCX)
