# Supplementary material for: One-year mortality and morbidities of severe fever with thrombocytopenia syndrome compared with other diseases: A nationwide cohort study in South Korea
Source: PLoS Negl Trop Dis. 2024 Jun 14;18(6):e0012253. doi: 10.1371/journal.pntd.0012253 (PMC11210842; doi:10.1371/journal.pntd.0012253)
Supplement: S1 Table — (DOCX) [file pntd.0012253.s001.docx]

**S1 Table. KCD-7, 8 codes for comorbidities**

| Comorbidity | | KCD-7,8 Codes | |
| --- | --- | --- | --- |
| Hypertension | | I10-I15 | |
| Diabetes mellitus | | E10-E14, O24, G63.2, H36.0 | |
| Stroke | | I60-63, I69 | |
| Heart failure | | I110, I130, I132, I255, I420, I429, I43, I50 | |
| Atrial fibrillation | | I48 | |
| Coronary artery occlusive disease | | I20-I25 | |
| Asthma | | J45-46 | |
| Chronic kidney disease | | N18-9, I12-3, Z992, E102, E112, E132, E142, N250, Z490, 491-2, Z940, E122, T861 | |
| Malignancy | | C00-C97, V027, V193-4 | |

Abbreviations: KCD, Korean Standard Classification of Diseases and Causes of Death
