# Supplementary material for: One-year mortality and morbidities of severe fever with thrombocytopenia syndrome compared with other diseases: A nationwide cohort study in South Korea
Source: PLoS Negl Trop Dis. 2024 Jun 14;18(6):e0012253. doi: 10.1371/journal.pntd.0012253 (PMC11210842; doi:10.1371/journal.pntd.0012253)
Supplement: S2 Table — (DOCX) [file pntd.0012253.s002.docx]

**S2 Table. Time from hospitalization to death in patients with SFTS or non-SFTS-related diseases**

|  | Group | Mean ± SD |
| --- | --- | --- |
| Death 0-365days | All | 54.6 ± 84.3 |
|  | SFTS | 20.8 ± 52.4 |
|  | Non-SFTS | 76.2 ± 93.3 |
| Death 0-30days | All | 8.1 ± 6.8 |
|  | SFTS | 7.0 ± 5.4 |
|  | Non-SFTS | 9.2 ± 7.9 |
| Death 31-365days | All | 142.8 ± 93.4 |
|  | SFTS | 128.6 ± 108.5 |
|  | Non-SFTS | 144.9 ± 91.3 |
