# Supplementary material for: One-year mortality and morbidities of severe fever with thrombocytopenia syndrome compared with other diseases: A nationwide cohort study in South Korea
Source: PLoS Negl Trop Dis. 2024 Jun 14;18(6):e0012253. doi: 10.1371/journal.pntd.0012253 (PMC11210842; doi:10.1371/journal.pntd.0012253)
Supplement: S3 Table — (DOCX) [file pntd.0012253.s003.docx]

**S3 Table. Time from discharge to events in patients with SFTS or non-SFTS-related diseases**

|  | Group | Mean ± SD |
| --- | --- | --- |
| Hospital admission | All | 80.6 ± 102.4 |
|  | SFTS | 60.2 ± 93.7 |
|  | Non-SFTS | 87.6 ± 104.3 |
| ER visit | All | 68.1 ± 93.6 |
|  | SFTS | 33.9 ± 67.5 |
|  | Non-SFTS | 88.5 ± 100.8 |
| Cardiovascular disease | All | 64.5 ± 79.7 |
|  | SFTS | 64.3 ± 85.6 |
|  | Non-SFTS | 59.2 ± 77.7 |
| Cerebrovascular event | All | 60.6 ± 79.5 |
|  | SFTS | 58.2 ± 84.5 |
|  | Non-SFTS | 61.1 ± 80.7 |
| ICU admission | All | 95.1 ± 88.9 |
|  | SFTS | 90.7 ± 80.0 |
|  | Non-SFTS | 96.5 ± 92.5 |
