# Supplementary material for: One-year mortality and morbidities of severe fever with thrombocytopenia syndrome compared with other diseases: A nationwide cohort study in South Korea
Source: PLoS Negl Trop Dis. 2024 Jun 14;18(6):e0012253. doi: 10.1371/journal.pntd.0012253 (PMC11210842; doi:10.1371/journal.pntd.0012253)

**S2 Fig.** **Age-specific Kaplan–Meier curves and hazard ratios for patients with SFTS or non-SFTS-related diseases for 31–365 days after hospitalization** (A) All age groups; (B) patients aged 20–49 years; (C) patients aged 50–59 years; (D) patients aged 60–69 years; (E) patients aged 70–79 years; and (F) patients aged 80–89 years.


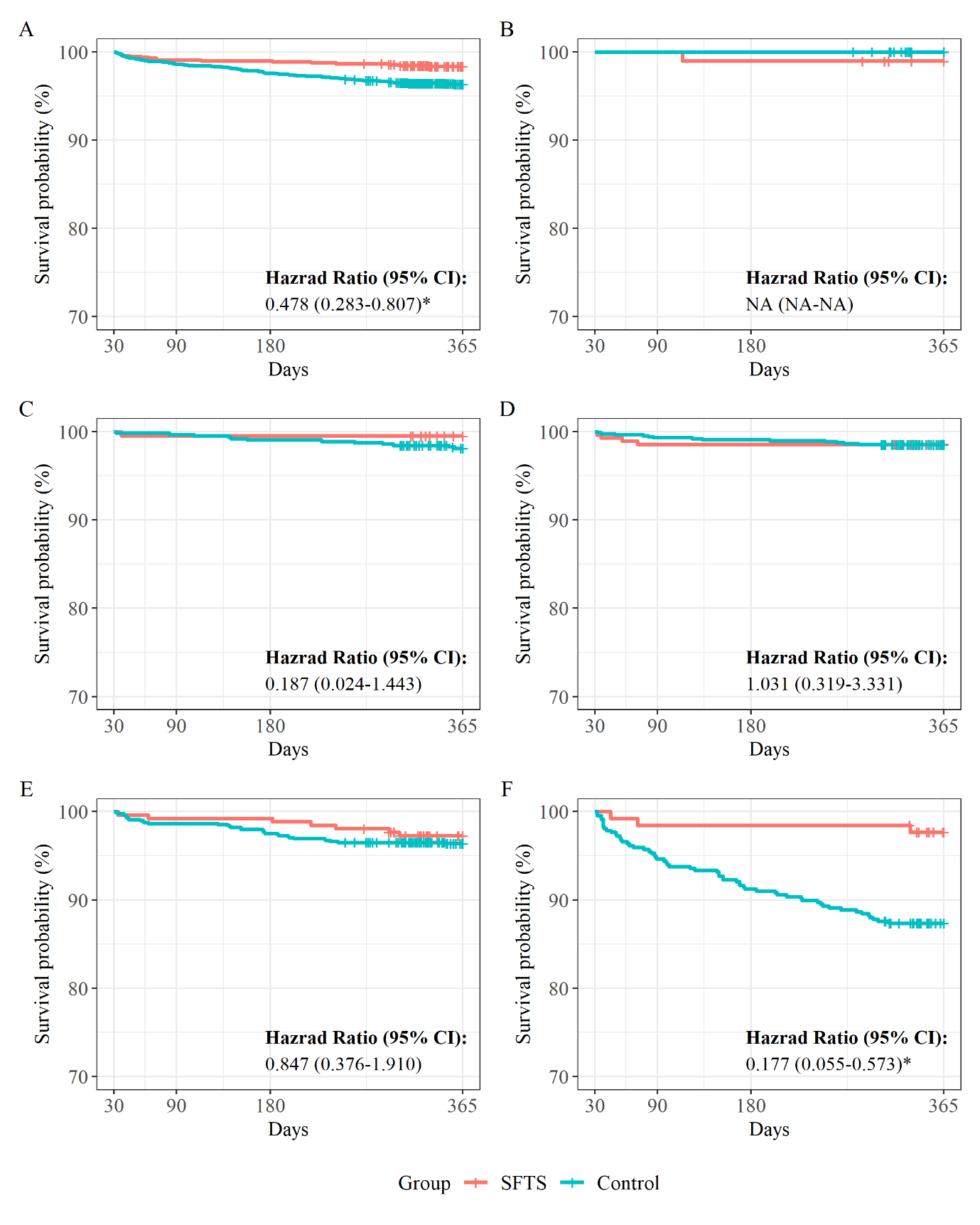

Supplement: S2 Fig — Age-specific Kaplan–Meier curves and hazard ratios for patients with SFTS or non-SFTS-related diseases for 31–365 days after hospitalization (A) All age groups; (B) patients aged 20–49 years; (C) patients aged 50–59 years; (D) patients aged 60–69 years; (E) patients aged 70–79 years; and (F) patients aged 80–89 years. (DOCX) [file pntd.0012253.s005.docx]
