# Supplementary material for: One-year mortality and morbidities of severe fever with thrombocytopenia syndrome compared with other diseases: A nationwide cohort study in South Korea
Source: PLoS Negl Trop Dis. 2024 Jun 14;18(6):e0012253. doi: 10.1371/journal.pntd.0012253 (PMC11210842; doi:10.1371/journal.pntd.0012253)

**S3 Fig.** **Cumulative incidence of post-discharge event during the one-year follow-up** in the (A) SFTS group at 0–30 days, (B) SFTS group at 31–365 days, (C) non-SFTS-related disease group at 0–30 days, and (D) non-SFTS-related disease group at days 31–365.


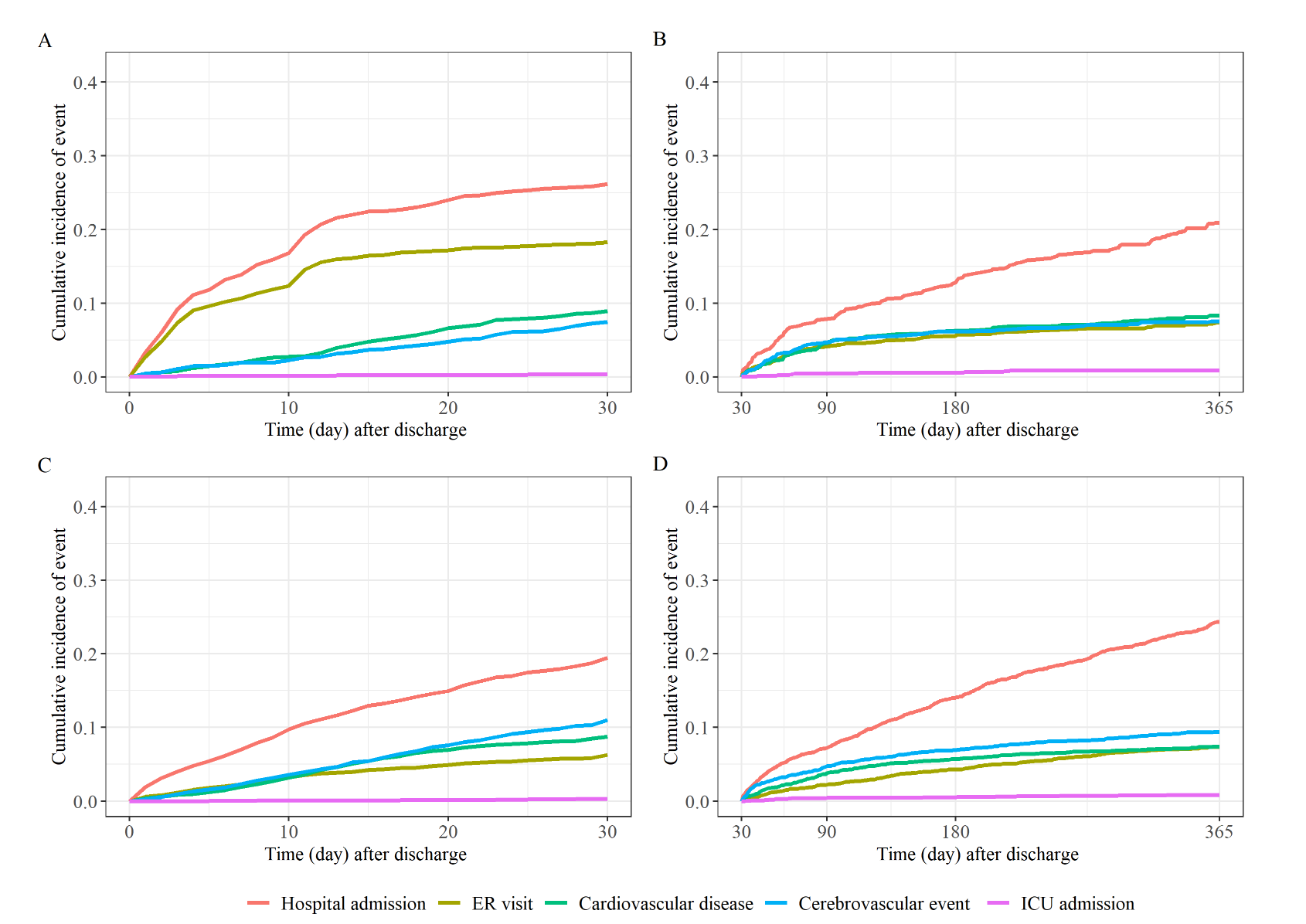

Supplement: S3 Fig — Cumulative incidence of post-discharge event during the one-year follow-up in the (A) SFTS group at 0–30 days, (B) SFTS group at 31–365 days, (C) non-SFTS-related disease group at 0–30 days, and (D) non-SFTS-related disease group at days 31–365. (DOCX) [file pntd.0012253.s006.docx]
