# Supplementary material for: One-year mortality and morbidities of severe fever with thrombocytopenia syndrome compared with other diseases: A nationwide cohort study in South Korea
Source: PLoS Negl Trop Dis. 2024 Jun 14;18(6):e0012253. doi: 10.1371/journal.pntd.0012253 (PMC11210842; doi:10.1371/journal.pntd.0012253)

**S4 Fig.** **Cumulative incidence of post-discharge event during the one-year follow-up** in patients with SFTS (A, C, E, G, and I) and non-SFTS patients (B, D, F, H, and J). (A, B) aged 20–49 years, (C, D) aged 50–59 years, (E, F) aged 60–69 years, (G, H) aged 70–79 years, (I, J) aged 80–89 years.


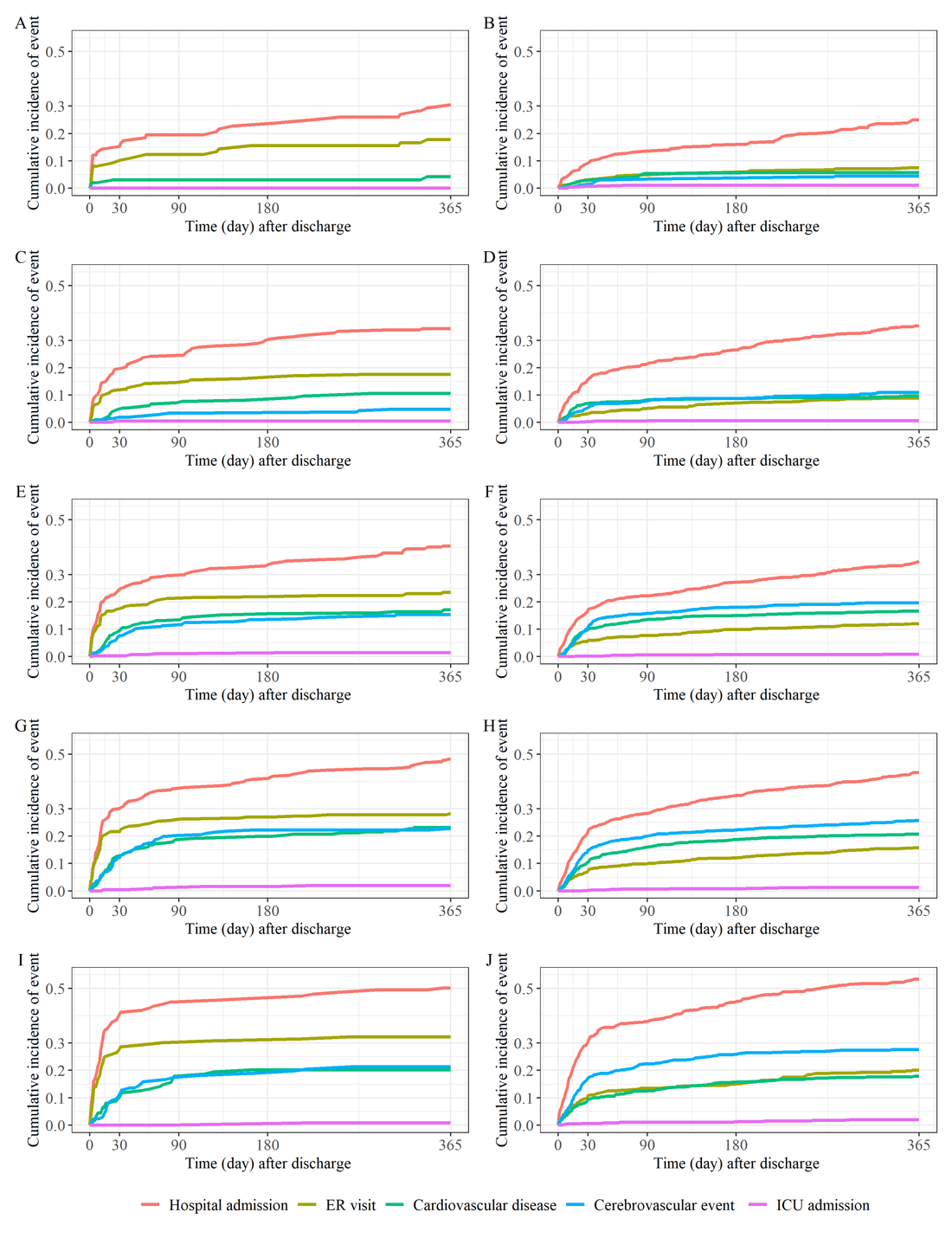

Supplement: S4 Fig — Cumulative incidence of post-discharge event during the one-year follow-up in patients with SFTS (A, C, E, G, and I) and non-SFTS patients (B, D, F, H, and J). (A, B) aged 20–49 years, (C, D) aged 50–59 years, (E, F) aged 60–69 years, (G, H) aged 70–79 years, (I, J) aged 80–89 years. (DOCX) [file pntd.0012253.s007.docx]
